# Effect of Intermediate Semiconducting TiO_x_ Thin Films on Nanoparticle-Mediated Electron Transfer: Electrooxidation of CO

**DOI:** 10.3390/nano12050855

**Published:** 2022-03-03

**Authors:** Aigerim Galyamova, Richard M. Crooks

**Affiliations:** Department of Chemistry, Center for Electrochemistry, Texas Materials Institute, The University of Texas at Austin, 105 E. 24th St., Stop A5300, Austin, TX 78712-1224, USA; aigerimg@utexas.edu

**Keywords:** electrochemistry, CO electrooxidation, thin films, gold nanoparticles, platinum nanoparticles, titanium dioxide

## Abstract

The concept of nanoparticle-mediated electron transfer (eT) across insulating thin films was elucidated theoretically by Allongue and Chazalviel in 2011. In their model, metal nanoparticles (NPs) are immobilized atop passivating, self-assembled monolayers (SAMs). They found that under certain conditions, related to the thickness of the SAM and the size of the NPs, efficient faradaic oxidation and reduction reactions could proceed at the NP surface. In the absence of NPs, however, eT was suppressed by the insulating SAM thin films. Allongue and Chazalviel concluded that, within certain bounds, eT is mediated by fast tunneling between the conductive electrode and the metal NPs, while the kinetics of the redox reaction are controlled by the NPs. This understanding has been confirmed using a variety of experimental models. The theory is based on electron tunneling; therefore, the nature of the intervening medium (the insulator in prior studies) should not affect the eT rate. In the present manuscript, however, we show that the theory breaks down under certain electrochemical conditions when the medium between conductors is an *n*-type semiconductor. Specifically, we find that in the presence of either Au or Pt NPs immobilized on a thin film of TiO_x_, CO electrooxidation does not proceed. In contrast, the exact same systems lead to the efficient reduction of oxygen. At present, we are unable to explain this finding within the context of the model of Allongue and Chazalviel.

## 1. Introduction

**Scheme 1:**
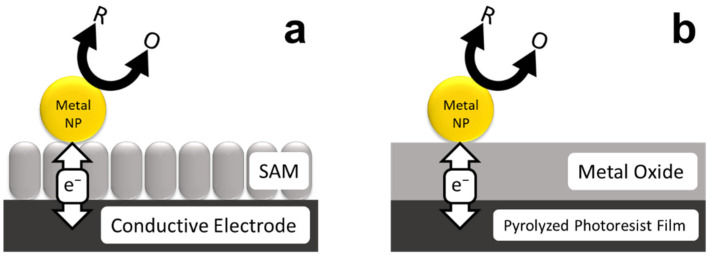
NP-mediated eT across insulating (**a**) SAM and (**b**) metal oxide thin films.

The concept of nanoparticle-mediated electron transfer (eT) across insulating thin films was elucidated theoretically by Allongue and Chazalviel in 2011 [1]. In their model, metal nanoparticles (NPs) are immobilized atop passivating, self-assembled monolayers (SAMs) (Scheme 1a). They found that under certain conditions, related to the thickness of the SAM and the size of the NPs, efficient faradaic oxidation and reduction reactions could proceed at the NP surface. In the absence of the NPs, however, eT was suppressed by the insulating SAM thin films. Allongue and Chazalviel concluded that, within certain bounds, eT is mediated by fast tunneling between the conductive electrode and the metal NPs, while the kinetics of the redox reaction are controlled by the NPs.

The foregoing theoretical findings were subsequently confirmed and expanded upon experimentally by Gooding and Paddon-Row, and Fermin [2,3]. Specifically, Fermin demonstrated long-distance electronic communication between AuNPs and SAM-modified Au electrode using Fe(CN)_6_^3−/4−^ as a redox probe [2]. Similarly, Gooding, Paddon-Row, and co-workers studied the electrochemical response of Ru(NH_3_)_6_^3+/2+^ at Au electrodes modified with an SAM layer in the presence and absence of AuNPs [3]. Just as predicted by Allongue and Chazalviel, they found a suppression of faradaic current in the absence of the AuNPs, but full recovery when AuNPs were present on the surface of the SAM.

Most reports of NP-mediated eT have involved reversible redox couples such as Ru(NH_3_)_6_^3+/2+^ or Fe(CN)_6_^3−/4−^ at either SAM- or polymer-modified electrodes [4,5,6]. In contrast, our group has been studying electrocatalytic reactions at electrodes passivated with thin oxide layers modified with either PtNPs [7,8] or AuNPs [9,10]. This configuration is illustrated in Scheme 1b. In this case, the underlying electrode is a pyrolyzed photoresist film (PPF) [11] and the thin oxide layers are produced using atomic layer deposition (ALD). The purpose of these investigations has been to systematically study how strong metal–support interactions (SMSIs) affect electrocatalytic reactions.

In our first study, we validated the model shown in Scheme 1b using Al_2_O_3_, a metal oxide that does not engender SMSI [7,8]. Specifically, we tested the electrochemical activity of a construct we denote as PPF/Al_2_O_3_(2.5 nm)/PtNP, where 2.5 nm is the thickness of the intermediate oxide film. We examined both the reversible redox molecule Fc(MeOH)_2_ and the oxygen reduction reaction (ORR) at this interface. Importantly, the results showed that this experimental model system closely follows the theoretical model described by Allongue and Chazalviel for both reactions. Furthermore, we confirmed that Al_2_O_3_ has no effect on the ORR activity of the supported PtNPs.

In contrast to our study of Al_2_O_3_, we reported on a synergistic relationship between AuNPs and TiO_x_ (2.8 nm thick, *x* = 1.9; and 2.0) supports towards the ORR [10]. Specifically, we observed a reproducible ~100 mV positive shift in the onset potential, and an increase in the effective number of electrons transferred during the ORR, at TiO_x_-supported AuNPs compared with AuNPs in the absence of support effects. Importantly, these results were found to be in quantitative agreement with density functional theory (DFT) calculations. In this study, the theoretical calculations were carried out using just one descriptor: the binding energy of the adsorbed OH intermediate (OH*). The results showed that a synergetic relationship exists between AuNPs and the TiO_x_ support due to a partial charge transfer from oxygen vacancies in the TiO_x_ to the AuNPs. This change in charge distribution is responsible for optimizing the OH* binding energy on the AuNPs; hence, the ORR enhancement. Experimentally, X-ray photoelectron spectroscopy (XPS) confirmed the presence of increased electron density on the AuNPs when they were in direct contact with the TiO_x_ support. This successful collaboration between theory and experiment encouraged us to apply our model to a more complex reaction.

Following the ORR study, we wished to expand the complexity of the DFT calculations to include a reaction requiring two descriptors and then verify the degree of theoretical efficacy. Preliminary calculations suggested that the CO electrooxidation reaction would be a good target, because its activity at AuNPs supported on TiO_x_ thin films is predicted to increase. Accordingly, we constructed a model system similar to that used for the foregoing ORR study. The results presented herein are surprising (but reproducible and consistent). Specifically, we observed little or no activity for CO electrooxidation. Detailed control experiments indicate the reason for this is that electrons are unable to tunnel through the TiO_x_ layer at positive potentials. This finding calls into question the universality of the experimental model shown in Scheme 1.

## 2. Materials and Methods

### 2.1. Chemicals and Materials

The following chemicals were used as received: [Ru(NH_3_)_6_]Cl_3_ (98%,Acros Organics, Morris Plains, NJ, USA); 1,1′-ferrocenedimethanol (Fc(MeOH)_2_, 98%, Acros Organics, Morris Plains, NJ, USA); KNO_3_ (certified, Fisher Scientific, Waltham, MA, USA); NaOH (1 M, Fisher Scientific, Waltham, MA, USA); HClO_4_ (+70%, ultrapure grade, J. T. Baker, Phillipsburg, NJ, USA); o-H_3_PO_4_ (85%, certified ACS, Fisher Scientific, Waltham, MA, USA); KH_2_PO_4_, K_2_HPO_4_, K_3_PO_4_ (certified, Fisher Scientific, Waltham, MA, USA); NaBH_4_ (99.99% trace metals basis, Sigma-Aldrich, Saint Louis, MO, USA); CuSO_4_ (98%, pure, anhydrous, Acros Organics, Morris Plains, NJ, USA); HAuCl_4_·3H_2_O (99.9% trace metals basis, Sigma-Aldrich, Saint Louis, MO, USA); K_2_PtCl_4_ (99.99% trace metals basis, Acros Organics, Morris Plains, NJ, USA), tetrakis(dimethylamino)titanium (IV) (TDMAT, 99%, STREM Chemicals, Inc., Newburyport, MA, USA), and trimethylaluminum (TMA, 98%, STREM Chemicals, Inc., Newburyport, MA, USA). The following compressed gases were purchased from Praxair (Austin, TX, USA): high-purity CO (99.5%), high-purity O_2_ (99.995%), high-purity Ar (99.998%), and forming gas (5% H_2_/95% N_2_).

Sixth-generation, amine-terminated (G6NH_2_) and hydroxy-terminated (G6OH) poly(amidoamine) dendrimers were purchased as 9.00 wt% and 12.98 wt% methanol solutions, respectively, from Dendritech, Inc. (Midland, MI, USA). For the dendrimer-encapsulated NP (DEN) synthesis, methanol was removed from the stock solution under vacuum and the dendrimers were reconstituted in water. UHPLC-grade water from Sigma-Aldrich (Saint Louis, MO, USA) was used to reconstitute the dendrimers, to synthesize DENs, and to prepare all aqueous solutions.

For PPF fabrication, fused quartz slides (GE 124, 3” × 1” × 1 mm) were purchased from Technical Glass Products, Inc. (Painesville Township, OH, USA), photoresist (AZ 1518), and photoresist developer (AZ 400 K, 1:4) were purchased from Integrated Micro Materials (Argyle, TX, USA).

### 2.2. Fabrication of Pyrolyzed Photoresist Film (PPF) Electrodes

PPF electrodes were fabricated by following a previously reported procedure that can be found elsewhere [10].

### 2.3. Deposition of ALD Thin Films on PPF Electrodes

The metal oxide thin films, TiO_x_ (*x* = 1.9; and 2.0) and Al_2_O_3_, were deposited using previously reported procedures [7,10]. Additional details can be found in the Supplementary Materials.

### 2.4. Synthesis and Immobilization of Au DENs

G6NH_2_(Au_147_) DENs were synthesized as follows using a previously published direct-reduction procedure [9,10]. First, 200 μL of a 100 μM aqueous G6NH_2_ dendrimer solution was added to 8.65 mL of vigorously stirred UHPLC water. Second, 147 μL of 20.0 mM HAuCl_4_ was pipetted dropwise into the diluted dendrimer solution. Third, within 2 min after adding the first drop of the HAuCl_4_ solution, a ~67-fold molar excess of NaBH_4_ (in 1.0 mL of 0.30 M NaOH) was added. Then, the reaction mixture was stirred overnight in air to deactivate excess NaBH_4_. This synthesis has previously been shown to produce AuNPs with an average size of 1.6 ± 0.2 nm (Figure S1 in the Supplementary Materials) [12,13].

Prior to immobilizing the G6NH_2_(Au_147_) DENs onto the PPF/TiO_x_ supports, the pH of the DENs solution was adjusted to ~3.2 using 1.0 M HClO_4_. We previously showed that this adjustment does not alter the size distribution of the AuNPs [10]. The G6NH_2_(Au_147_) DENs were immobilized by immersing the PPF/TiO_x_ supports in the DENs solution for 90 min. After immobilization, the modified supports (PPF/TiO_x_/G6NH_2_(Au_147_)) were gently rinsed with UHPLC water and dried under a flow of Ar. The modified supports were further air-dried in the lab for at least 1 h prior to the use.

### 2.5. Synthesis and Immobilization of Pt DENs

G6OH(Pt_55_) DENs were prepared as follows using a previously published indirect galvanic exchange method [7]. First, 1.0 mL of 100 μM of an aqueous G6OH dendrimer solution was added to 8.68 mL of moderately stirred UHPLC water. This solution was kept under an Ar atmosphere for the duration of the synthesis. Second, 0.275 mL of 20.0 mM CuSO_4_ was pipetted dropwise into the diluted dendrimer solution and it was allowed to equilibrate for 15 min. Then, 32.7 μL of 1.0 M NaBH_4_ was pipetted dropwise into the G6OH-(Cu^2+^)_55_ DEN precursor solution, and the reaction mixture was stirred for 50 min. Subsequently, the pH of the solution was adjusted to 3.0 using 1.0 M HClO_4_. Finally, 0.550 mL of 10.0 mM K_2_PtCl_4_ was pipetted dropwise into the G6OH(Cu_55_) solution. The final reaction mixture was stirred for 60 min to allow complete galvanic exchange between Cu^0^ and Pt^2+^ to occur. This synthesis produced PtNPs with an average size of 1.3 ± 0.2 nm (Figure S2 in the Supplementary Materials) [7].

The G6OH(Pt_55_) DENs were immobilized by immersing the PPF/TiO_x_ or PPF/Al_2_O_3_ supports in the DENs solution for 45 min. After the immobilization, the modified supports (PPF/TiO_x_/G6OH(Pt_55_) or PPF/Al_2_O_3_/G6OH(Pt_55_)), were rinsed with UHPLC water and dried under flowing Ar. The modified supports were further air-dried in the lab for at least 1 h prior to use.

### 2.6. UV/O_3_ Method for Decomposition of G6NH_2_ and G6OH Dendrimers

We previously reported a procedure for decomposing dendrimers using a UV/O_3_ process [8,9,10]. The resulting PPF/TiO_x_/Au_147_, PPF/TiO_x_/Pt_55_, and PPF/Al_2_O_3_/Pt_55_ (Figure S3 in the Supplementary Materials) supports were equilibrated in an ambient atmosphere for at least 45 min before use.

### 2.7. Structural Characterization

XPS measurements were performed using a Kratos Axis Ultra DLD spectrometer (Chestnut Ridge, NY, USA). The samples were analyzed according to a previous report from our lab [10]. Briefly, the spectra were collected using an Al K_α_ source, 0.10 eV step size, and 20 eV band pass energy. CasaXPS (version 2.3.19, Casa Software, Teignmouth, UK) was used for peak fitting and quantitative data analysis. Binding energies (BEs) were calibrated using the C 1s line of PPF (284.50 eV) [14,15]. The specific method used for peak fitting and the oxygen vacancy calculation for the TiO_x_ thin films have been described previously [10]. Likewise, electronic interactions between TiO_x_ and Al_2_O_3_ thin-film-supported metal NPs have been extensively characterized in our related studies [7,8,9,10].

Ellipsometric thickness measurements were performed using a J. A. Woollam M-2000 D spectroscopic ellipsometer (Lincoln, NE, USA). Data were collected between 45° and 65° with 5° increments and a 10 s dwell time at each step. The specific model that was created for the data analysis has been described previously [10].

Transmission electron microscopy (TEM) images were collected using a JEOL-2010F TEM (JOEL USA Inc., Peabody, MA, USA) having a point-to point resolution of 0.2 nm. For this, 2.0 μL of the DENs solution was pipetted onto a carbon-mesh-over-Cu TEM grid (Electron Microscopy Sciences, Hatfield, PA, USA). The samples were left to dry overnight in the ambient laboratory atmosphere prior to the analysis. We have previously described the topography and the crystallinity of the TiO_x_ and Al_2_O_3_, ALD thin films [7,8,10].

### 2.8. Electrochemical Characterization

A CHI 700E bipotentiostat, a Hg/Hg_2_SO_4_ reference electrode, and a glassy carbon rod counter electrode were used for all electrochemical measurements (CH Instruments Inc., Austin, TX, USA). These measurements were performed in a Teflon electrochemical cell which constrained the geometric area of the working electrode to 12.4 mm^2^ [7].

Prior to the electrocatalytic experiments, the extent of passivation and the stability of the PPF/TiO_x_ and PPF/Al_2_O_3_ supports were determined using 1.0 mM Fc(MeOH)_2_ and 0.10 M KNO_3_. All electrocatalytic experiments were performed using electrocatalysts from which dendrimer had been removed. Note, however, that there is no significant difference in CO electrooxidation activity on electrocatalysts with and without the dendrimer (Figure S4 in the Supplementary Materials). The high degree of stability of electrocatalysts containing metal NPs, PPF/TiO_x_/Au_147_ and PPF/Al_2_O_3_/Pt_55_, have previously been described [7,8,9,10].

Prior to the CO electrooxidation experiments, all modified supports were electrochemically cleaned in an Ar-saturated, 0.10 M HClO_4_ solution. During the cleaning of the supports with AuNPs on the surface, the electrode potential was scanned ten times between −0.20 and 0.87 V at 0.050 V/s. During the cleaning of the supports with PtNPs on the surface, the electrode potential was scanned ten times between −0.65 and 0.63 V at 0.050 V/s.

Following electrochemical cleaning, CO electrooxidation using the supports with AuNPs on the surface was performed in a CO-saturated, 0.10 M NaOH solution. CO electrooxidation on the supports with PtNPs on the surface were performed in a CO-saturated, 0.10 M HClO_4_ solution.

The rectifying behavior of the PPF/TiO_x_ supports was studied using 1.0 mM [Ru(NH_3_)_6_]Cl_3_ in an Ar-saturated, 0.10 M phosphate-buffered solutions.

## 3. Results and Discussion

### 3.1. Properties of TiO_x_ (x = 1.9; and 2.0) Thin Films

Ellipsometry was used to measure the TiO_x_ film thicknesses as a function of the number of ALD cycles performed. The growth rate of the films was 0.060 ± 0.002 nm/cycle with a nucleation delay [16] of 12 ALD cycles. For the experiments discussed here, the TiO_x_ films were deposited using 50 ALD cycles, which is equivalent to ~2.3 nm in total thickness. The degree of PPF electrode passivation of these TiO_x_ (2.3 nm)-coated PPF electrodes was determined using cyclic voltammetry (CV). For these experiments, an aqueous solution containing 1.0 mM Fc(MeOH)_2_ and 0.10 M KNO_3_ was used. Figure S5 in the Supplementary Materials compares CVs for PPF-only, PPF/TiO_1.9_(2.3 nm), and PPF/TiO_2.0_(2.3 nm) working electrodes. The results indicate near-complete passivation of faradaic electrochemistry for the two PPF/TiO_x_ films, which is consistent with our previous findings [10].

### 3.2. CO Electrooxidation at PPF-Supported Au_147_ NPs

**Figure 1:**
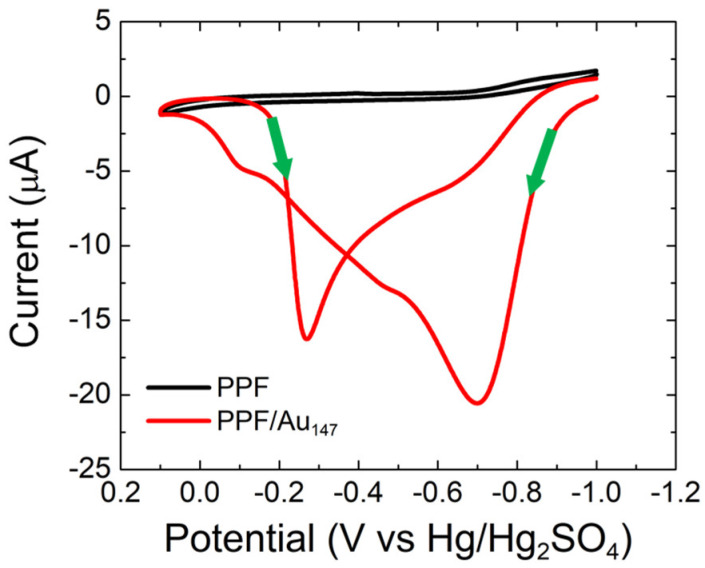
CVs obtained for CO electrooxidation on bare PPF and PPF/Au_147_ electrodes. The solutions contained aqueous, CO-saturated 0.10 M NaOH. The electrode potential was scanned between −1.0 and 0.10 V at 0.050 V/s. The experiments were carried out in triplicate for each electrode configuration using independently prepared electrodes to ensure reproducibility.

Figure 1 shows CVs for PPF-only and PPF/Au_147_ electrodes in CO-saturated, 0.10 M NaOH. The alkaline environment was chosen for this experiment because AuNPs smaller than 2.5 nm exhibit little to no CO electrooxidation activity in aqueous acidic solutions [17]. The CV for the PPF-only electrode is inactive for CO electrooxidation. In contrast, a complex CV is observed when Au_147_ NPs are present atop the PPF surface. Specifically, anodic currents are observed in both the forward and reverse scans. This is a consequence of electrocatalyst deactivation at more positive potentials and subsequent reactivation at less positive potentials, as discussed subsequently [17,18,19]. This behavior is consistent with the Langmuir–Hinshelwood mechanism [19].

Focusing on the forward scan of the PPF/Au_147_ CV, a broad wave corresponding to CO electrooxidation is observed. It consists of a main peak at −0.70 V, and shoulders at −0.47 V and −0.11 V. These three features arise from heterogeneous binding sites on the AuNP electrocatalyst [19]. The maximum CO electrooxidation activity occurs at −0.70 V. As the scan continues, CO is depleted at the electrode surface, and OH* begins to poison the electrocatalyst surface due to increased binding energy (reaching a maximum at 0.10 V) [19].

Upon scan reversal, the OH* binding strength decreases at less positive potentials, thereby making it possible for CO to co-bind to the Au surface. Accordingly, CO electrooxidation continues via the Langmuir–Hinshelwood mechanism. A corresponding broad, reverse wave is present with a main peak at −0.27 V and a shoulder at −0.68 V. The CO electrooxidation current is lower in the reverse direction due to depletion of bulk CO in the vicinity of the electrode surface. Regardless of these details, the key point of this discussion is that the Au_147_ NPs are active toward CO electrooxidation, and the voltammetry is consistent with literature reports [19].

### 3.3. CO Electrooxidation at Au_147_ NPs on TiO_x_ Thin Films

**Figure 2:**
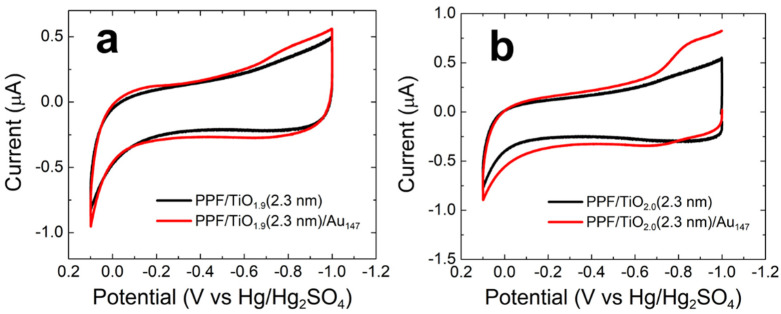
CVs obtained for CO electrooxidation on (**a**) PPF/TiO_1.9_(2.3 nm)/Au_147_ and (**b**) PPF/TiO_2.0_(2.3 nm)/Au_147_ electrodes. The specific electrode configurations are given in the legends. The solutions contained aqueous, CO-saturated 0.10 M NaOH. The electrode potential was scanned between −1.0 and 0.10 V at 0.050 V/s. The experiments were carried out in triplicate for each electrode configuration using independently prepared electrodes to ensure reproducibility.

Figure 2a shows CVs for PPF/TiO_1.9_(2.3 nm) and PPF/TiO_1.9_(2.3 nm)/Au_147_ in CO-saturated, 0.10 M NaOH. The CV for the PPF/TiO_1.9_ electrode reveals the background activity for the TiO_1.9_ thin film for the same CO electrooxidation conditions reported in the previous section. Specifically, an increase in anodic current is observed at −0.10 V due to the oxygen evolution reaction [20]. No other prominent faradaic activity is detected at PPF/TiO_1.9_ supports under alkaline conditions.

Surprisingly, the CV for the PPF/TiO_1.9_(2.3 nm)/Au_147_ is almost identical to that of the AuNP-free electrode, indicating little or no CO electrooxidation activity. However, there is a slight increase in cathodic current around −0.8 V in the reverse scan that only appears when Au_147_ NPs are present. We associate this small peak with the AuNP-catalyzed ORR. No other significant level of faradaic activity is observed.

Figure 2b shows CVs analogous to those discussed above, but for the oxidized TiO_2.0_(2.3 nm) thin film. The results are consistent with those shown in Figure 2a, with just a slight increase in the ORR current compared with the control experiment, for the PPF/TiO_2.0_(2.3 nm)/Au_147_ electrode.

We investigated the possibility of electrocatalyst deactivation under our experimental conditions. Specifically, immediately following the CO electrooxidation measurements, the electrocatalyst used to obtain the data in Figure 2a was tested for ORR activity (Figure S6 in the Supplementary Materials). The results show that significant ORR activity is observed under both acidic and alkaline conditions. Importantly, the ORR onset potential (~0.1 V vs. RHE), which we define as the potential corresponding to 10% of the ORR peak current, matches the value we reported previously [10]. This indicates that the electrocatalyst is stable and does not experience deactivation during CO electrooxidation experiments.

We also studied how the TiO_x_ thin film thickness affects the CO electrooxidation activity of PPF/TiO_x_/Au_147_ electrocatalysts (Figure S7 in the Supplementary Materials). The results show that no significant CO electrooxidation activity is observed for TiO_x_ thin films having thicknesses ranging from 1.1 to 2.9 nm.

The foregoing results are puzzling, because they do not follow the current understanding of NP-mediated eT across insulating thin films (Scheme 1). Accordingly, we continued our study by testing whether this surprising behavior is only observed for TiO_x_/AuNP by investigating CO electrooxidation activity at TiO_x_/PtNP.

### 3.4. CO Electrooxidation at PPF-Supported Pt_55_ NPs

**Figure 3:**
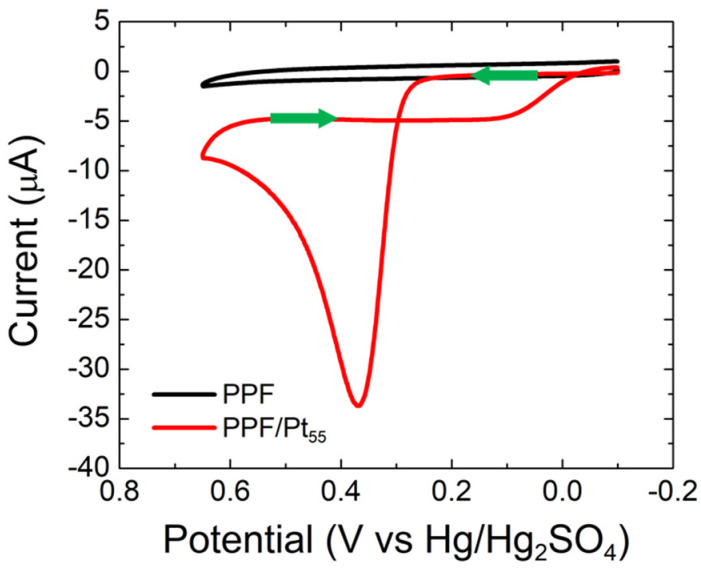
CVs obtained for CO electrooxidation on bare PPF and PPF/Pt_55_ electrodes. The solutions contained aqueous, CO-saturated 0.10 M HClO_4_. The electrode potential was scanned between –0.10 and 0.65 V at 0.050 V/s. The experiments were carried out in triplicate for each electrode configuration using independently prepared electrodes to ensure reproducibility.

Figure 3 shows CVs for PPF-only and PPF/Pt_55_ electrodes in CO-saturated, 0.10 M HClO_4_. We used acidic electrolytes for this experiment, rather than basic electrolytes, for two reasons. First, the CO electrooxidation at Pt catalysts in acidic media has been extensively characterized [21,22,23,24,25]. Second, key experiments involving Al_2_O_3_/PtNP supports, which are discussed later, require the use of acidic media due to the instability of Al_2_O_3_ in bases [26].

The CV for the PPF-only electrode indicates no CO electrooxidation activity for PPF under aqueous acidic conditions. In contrast, well-defined CO electrooxidation activity is present when Pt_55_ NPs are immobilized atop the PPF surface. Specifically, anodic currents are present in both the forward and reverse scans, with the current in the forward scan being ~10× higher than the reverse scan. This behavior is consistent with the accepted Langmuir–Hinshelwood mechanism for CO electrooxidation at Pt catalysts [21,22,23]. Here, the reactant-pair is CO* and OH*, where OH* originates from water oxidation at the electrode (rather than being present at high concentrations in the electrolyte, as for the AuNP case) [24].

Focusing on the forward scan of the PPF/Pt_55_ CV, a single peak at 0.37 V, corresponding to CO electrooxidation, is observed. This peak appears broader and is ~200 mV more positive compared with that observed for bulk Pt [21,23,24,25]. This is due to the increased heterogeneity of binding sites, and hence, the CO binding energies, on PtNPs compared with bulk Pt [23]. As the scan continues, the current decreases due to the depletion of CO in the vicinity of the electrode and the surface poisoning effect of an increase in the OH* binding energy at more positive potentials [25].

Upon scan reversal, the electrocatalyst surface is reactivated due to decreased OH* binding energy. However, the depletion of CO near the electrode surface leads to a lower anodic current. The key point of this discussion is that the Pt_55_ NPs are active toward CO electrooxidation, and the voltammetry is consistent with reports in the literature [21,22,23,24,25].

### 3.5. CO Electrooxidation at Pt_55_ NPs on TiO_x_ Thin Films

**Figure 4:**
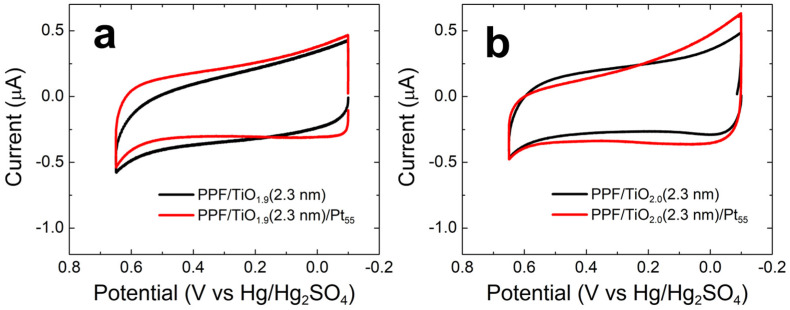
CVs obtained for CO electrooxidation on (**a**) PPF/TiO_1.9_(2.3 nm)/Pt_55_ and (**b**) PPF/TiO_2.0_(2.3 nm)/Pt_55_ electrodes. The specific electrode configurations are given in the legends. The solutions contained aqueous, CO-saturated 0.10 M HClO_4_. The electrode potential was scanned between –0.10 and 0.65 V at 0.050 V/s. The experiments were carried out in triplicate for each electrode configuration using independently prepared electrodes to ensure reproducibility.

Figure 4a shows CVs for PPF/TiO_1.9_(2.3 nm) and PPF/TiO_1.9_(2.3 nm)/Pt_55_ in CO-saturated, 0.10 M HClO_4_. The CV for the PPF/TiO_1.9_ electrode reveals the background activity for the TiO_1.9_ thin film for the same CO electrooxidation conditions discussed in the previous section. The results indicate a primarily capacitive current with a slight indication of anodic current at 0.65 V arising from the oxygen evolution reaction [20] and a slight cathodic current at −0.10 V from the hydrogen evolution reaction (HER) [27]. As for the AuNP results, the CV for the PPF/TiO_1.9_(2.3 nm)/Pt_55_ is almost identical to that of the PtNP-free electrode, indicating little or no CO electrooxidation activity. Figure 4b shows that CVs analogous to those for the TiO_1.9_ thin films are very similar when the film is fully oxidized to TiO_2.0_.

Comparison of the results for the TiO_x_/AuNP and TiO_x_/PtNP thin films suggests that the semiconducting nature of the TiO_x_ films could be responsible for the absence of CO electrooxidation. To test this hypothesis, we replaced TiO_x_ with Al_2_O_3_ (a dielectric with a bandgap of ~7 eV) [28] and investigated the resulting Al_2_O_3_/PtNP supports for CO electrooxidation. The outcome of these experiments is discussed next.

### 3.6. Properties of Al_2_O_3_ Thin Films

The properties of the Al_2_O_3_ thin films used in this part of the study were characterized exactly as for the TiO_x_ thin films. Ellipsometry revealed that the Al_2_O_3_ films grew at a rate of 0.081 ± 0.003 nm/cycle. For the experiments discussed here, the Al_2_O_3_ films were deposited using 28 ALD cycles, which is equivalent to ~2.3 nm in total thickness. The degree of PPF electrode passivation of these Al_2_O_3_(2.3 nm)-coated PPF electrodes was determined using the same technique and conditions as for the TiO_x_ films. Figure S8 in the Supplementary Materials compares CVs for PPF-only and PPF/Al_2_O_3_(2.3 nm) working electrodes. The results indicate the near-complete passivation of faradaic electrochemistry for the PPF/Al_2_O_3_ films, which is consistent with our previous findings [7,8].

### 3.7. CO Electrooxidation at Al_2_O_3_ Thin Film-Supported Pt_55_ NPs

**Figure 5:**
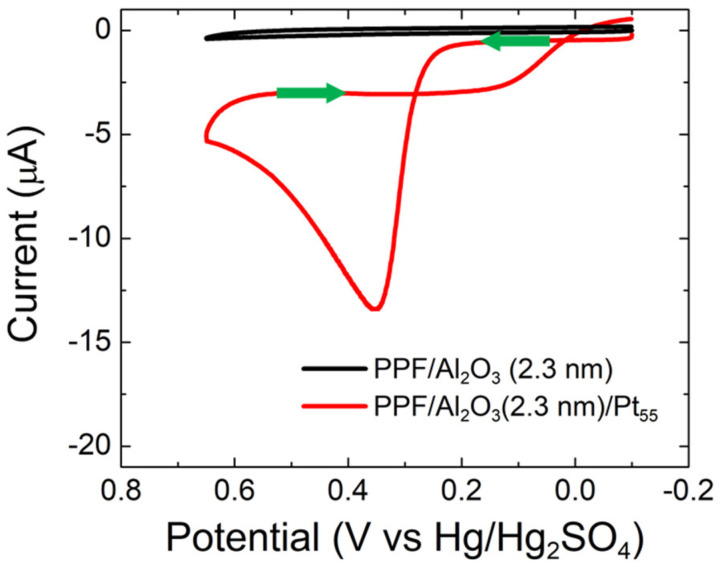
CVs obtained for CO electrooxidation on PPF/Al_2_O_3_(2.3 nm) and PPF/Al_2_O_3_(2.3 nm)/Pt_55_ electrodes. The solutions contained aqueous, CO-saturated 0.10 M HClO_4_. The electrode potential was scanned between –0.10 and 0.65 V at 0.050 V/s. The experiments were carried out in triplicate for each electrode configuration using independently prepared electrodes to ensure reproducibility.

Figure 5 shows CVs for PPF/Al_2_O_3_(2.3 nm) and PPF/Al_2_O_3_(2.3 nm)/Pt_55_ electrodes in CO-saturated, 0.10 M HClO_4_. The CV for PPF/Al_2_O_3_(2.3 nm) indicates that there is no CO electrooxidation activity at PtNP-free supports. In contrast, significant CO electrooxidation activity is present when Pt_55_ NPs are immobilized atop the PPF/Al_2_O_3_ supports. Importantly, the CO electrooxidation peak position (0.37 V) is the same as that observed at Al_2_O_3_-free PPF/Pt_55_ (Figure 3). Furthermore, the shapes of the CVs for PPF/Pt_55_ and PPF/Al_2_O_3_(2.3 nm)/Pt_55_ are similar. This indicates that Al_2_O_3_ thin films do not interfere with the CO electrooxidation activity of Pt_55_ NPs. This result is important because it demonstrates that under CO electrooxidation conditions, our model system (Scheme 1b) follows the theoretical expectation for NP-mediated eT. We have also investigated how Al_2_O_3_ thin film thickness affects the CO electrooxidation activity of PPF/Al_2_O_3_/Pt_55_ electrocatalysts (Figure S9 in the Supplementary Materials). CO electrooxidation activity is fully suppressed when 3.0 nm thick Al_2_O_3_ thin films are used to construct the electrocatalyst. This behavior is consistent with the model system proposed by Allongue and Chazalviel, where NP-mediated eT is expected to be hindered at thicknesses in the order of 3 nm for nanoparticles in the size range reported herein [1]. These findings confirm that the TiO_x_ thin films, rather than other components of the model system, are responsible for the absence of CO electrooxidation activity. Accordingly, we next focus our attention on the reason for this observation.

### 3.8. Electrochemical Characterization of TiO_x_ Thin Film Rectifying Behavior

**Figure 6:**
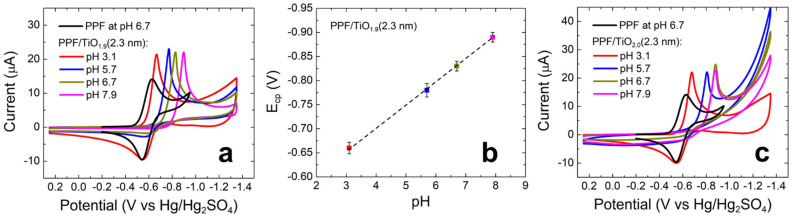
CVs obtained using (**a**) PPF/TiO_1.9_(2.3 nm) and (**c**) PPF/TiO_2.0_(2.3 nm) electrodes. The solutions contained aqueous 1.0 mM [Ru(NH_3_)_6_]Cl_3_ and 0.10 M phosphate buffer at the pHs indicated in the legends. The electrode potential was scanned between 0.25 and −1.35 V at 0.050 V/s. The experiments were carried out in triplicate for each electrode configuration using independently prepared electrodes to ensure reproducibility. (**b**) Plot showing the relationship between *E_cp_* and pH for the indicated working electrode configuration. The error bars in (**b**) represent the standard deviations from the mean for three measurements that were carried out using independently prepared electrodes.

Figure 6a shows CVs for bare PPF (black) and PPF/TiO_1.9_(2.3 nm) working electrodes in Ar-saturated 1.0 mM [Ru(NH_3_)_6_]Cl_3_ (E° = −0.54 V vs. Hg/Hg_2_SO_4_) [29] and 0.10 M phosphate buffer in the pH conditions specified in the legend. The purpose of this experiment was to test whether the TiO_x_ thin films exhibited the distinct rectifying and flat-band potential behavior that has previously been reported for defect-rich SnO_2_ and TiO_2_ ALD thin films [30,31].

The CV obtained using the bare PPF electrode (black) reveals reversible electroactivity with well-defined reduction and oxidation peaks at −0.63 and −0.54 V, respectively. The CVs obtained using the PPF/TiO_1.9_(2.3 nm) working electrode reveal the following features as a function of pH. For the negative-going scan at pH 3.1 (red), there is a sharp cathodic peak at −0.66 V, background activity between −0.80 and −1.2 V (due to the presence of phosphates, Figure S10 in the Supplementary Materials), and a slight cathodic current at −1.3 V arising from the HER [27]. Upon scan reversal, an anodic peak is present at −0.55 V. At pH 5.7 (blue), the sharp cathodic peak moves negative to −0.78 V, and the greatly attenuated anodic peak is present at −0.57 V. At higher pHs, the sharp cathodic peak continues to shift negative (Figure 6b). The anodic peak becomes indistinguishable from the background current at the higher pHs.

The results shown in Figure 6a,b lead to the following three conclusions. First, PPF/TiO_1.9_ working electrodes exhibit strong rectifying behavior towards Ru(NH_3_)_6_^3+^ reduction, which manifests as a significantly sharper cathodic peak compared with that obtained using bare PPF. Second, there is strong correlation between pH and the position of the cathodic peak that follows the relationship expressed in Equation (1) [31].

(1) $$E_{FB}=E_{BE}-0.059*pH$$

Here, *E_FB_* is the flat-band potential (the potential at which a semiconductor acts as a conductor) and *E_BE_* is the band-edge position of a semiconductor [29,31]. This equation indicates that for every unit pH change, there is a 59 mV shift in the flat-band potential. As shown by the plot in Figure 6b, we observe a linear relationship between *E_CP_*, the cathodic peak position, and pH with a 53 ± 6 mV slope. Third, as the pH increases, the anodic faradaic current decreases and completely disappears at pH 7.9. This decrease at the high pHs is a consequence of Equation (1). Specifically, at high pH, the flat-band potential of TiO_1.9_ is far from the standard potential of Ru(NH_3_)_6_^3+^ (E° = −0.54 V). For example, at pH 7.9 (pink), the TiO_1.9_ flat-band potential lies outside of the potential range where reduced Ru(NH_3_)_6_^3+^ can be re-oxidized. As a result, no anodic current is observed.

Figure 6c shows CVs for the same experiment discussed in the previous paragraph, but for a fully oxidized TiO_2.0_ thin film. With fairly minor differences in peak positions and the magnitude of the HER current, this set of CVs is very similar to those in Figure 6a. Note that the higher HER current for TiO_2.0_ is consistent with TiO_2.0_ thin film electrochemical activity that we have observed previously [10].

Notably, The TiO_2.0_ CVs do not follow Equation (1) as clearly as the CVs for TiO_1.9_. This is the most apparent at pHs 6.7 and 7.9, where the cathodic peaks appear at the same position (−0.88 V). In addition, as pH increases the anodic faradaic current at PPF/TiO_2.0_ diminishes faster than that of PPF/TiO_1.9_. This can be explained by the decrease in oxygen vacancies, or defect points, in the lattice structure of the TiO_2.0_ film [32].

The key point of the foregoing discussion is that the TiO_x_ (*x* = 1.9; and 2.0) thin films reveal intrinsic *n*-type semiconducting characteristics that control the electrochemical response of the PPF/TiO_x_ electrodes. This type of behavior has previously been reported by Grätzel and co-workers for defect-rich TiO_2_ ALD films [31]. They determined the flat-band potential of a 3 nm thick TiO_2_ ALD film to be ~0.10 V vs. RHE. This means that TiO_2_ ALD films will only conduct electrons at potentials more negative than 0.10 V vs. RHE. This finding is consistent with our observation of negligible CO electrooxidation activity at PPF/TiO_x_/Au_147_ and PPF/TiO_x_/Pt_55_ thin films. As discussed in the next section, however, the mystery remains as to why this semiconducting property should affect tunneling from Au_147_ or Pt_55_ NPs to PPF electrodes during CO electrooxidation. This result is particularly confounding because the ORR proceeds efficiently at identically prepared electrodes [10].

## 4. Summary and Conclusions

We [7,8,9,10] and others [2,3,4,5,6] have reported the experimental verification of the original theory [1] of enhanced tunneling between metal NPs and underlying electrodes separated by an intervening, nonconductive medium. We undertook the present study using TiO_x_ thin films for the electrooxidation of CO as a companion to related studies of the ORR [10]. In the latter case, we observed behavior consistent with the theory, but in the present case, no electroactivity was observed. This finding is perplexing because tunneling across TiO_x_ should be independent of the direction of the current.

To better understand the foregoing result, we designed a number of control experiments. For example, we considered that there might be something special about CO oxidation at AuNPs; therefore, we studied the same electrochemical reaction at PtNPs. In the presence of the TiO_x_ interlayer, no CO electrooxidation activity was observed for either metal. When the TiO_x_ interlayer is absent, however, CO electrooxidation proceeds in accordance with prior literature reports [17,18,19,21,22,23,24,25]. We also replaced the semiconducting TiO_x_ interlayer with insulating Al_2_O_3_ and found that CO electrooxidation proceeds as expected. This finding validates the NP-mediated eT mechanism (Scheme 1) for large bandgap dielectrics. This is an important result that has not previously been reported for a complex electrooxidation reaction for Scheme 1b configurations.

Finally, we also studied the electrochemistry of a model redox couple, Ru(NH_3_)_6_^3+/2+^_,_ using TiO_x_ interlayers and metal NPs. The results exhibited a pH dependency, which is perplexing because tunneling should not be affected by the nature of the medium intervening between conductors.

We plan to continue investigating the effect of semiconducting thin films on CO electrooxidation activity at metal NPs. Specifically, we are presently studying CO electrooxidation using intervening NiO thin films, which have *p*-type semiconducting properties. We hope that the results of these experiments will shed light on the present mystery. The results of those experiments will be reported in due course.

## Data Availability

The data is available on reasonable request from the corresponding author.

## Supplementary Materials

The following are available online at: https://www.mdpi.com/article/10.3390/nano12050855/s1, Deposition of ALD thin films on PPF electrodes, Figure S1: TEM micrograph and particle-size distribution histogram for G6NH_2_(Au_147_), Figure S2: TEM micrograph and particle-size distribution histogram for G6OH(Pt_55_), Figure S3: XPS spectra of G6NH_2_(Au_147_) and G6OH(Pt_55_) DENs before and after UV/O_3_ dendrimer removal procedure, Figure S4: CVs of CO electrooxidation on PPF/G6NH_2_(Au_147_) and PPF/G6OH(Pt_55_) before and after UV/O_3_ dendrimer removal procedure, Figure S5: CV overlay of Fc(MeOH)_2_ redox probe at PPF, PPF/TiO_1.9_ and PPF/TiO_2.0_ working electrodes, Figure S6: CVs of ORR on PPF/TiO_1.9_(2.3 nm)/Au_147_ in acidic and alkaline media, Figure S7: CVs of CO electrooxidation on PPF/TiO_x_/Au_147_ with varying TiO_x_ thin film thicknesses, Figure S8: CV overlay of Fc(MeOH)_2_ redox probe at PPF, and PPF/Al_2_O_3_ working electrodes, Figure S9: CVs of CO electrooxidation on PPF/Al_2_O_3_/Pt_55_ with varying Al_2_O_3_ thin film thicknesses, Figure S10: CV overlay of pH 3.1 phosphate buffer at PPF/TiO_1,9_, and PPF/TiO_2.0_ working electrodes. Refs. [7,10,27,33] cited in Supplementary Materials.
