# Supplementary material for: Effect of Intermediate Semiconducting TiOx Thin Films on Nanoparticle-Mediated Electron Transfer: Electrooxidation of CO
Source: Nanomaterials (Basel). 2022 Mar 3;12(5):855. doi: 10.3390/nano12050855 (PMC8912720; doi:10.3390/nano12050855)
Supplement: Supplementary file 1 [file nanomaterials-12-00855-s001.zip › nanomaterials-1538567-supplementary.pdf]

Supplementary Materials

Effect of intermediate semiconducting TiO_x thin films on nanoparticle-mediated electron transfer: electrooxidation of CO

Aigerim Galyamova and Richard M. Crooks

1 Deposition of ALD thin films on PPF electrodes.
2 TEM micrograph and particle-size distribution histogram for G6NH$_2$(Au$_{147}$), Figure S1.
3 TEM micrograph and particle-size distribution histogram for G6OH(Pt$_{55}$), Figure S2.
4 XPS spectra of G6NH$_2$(Au$_{147}$) and G6OH(Pt$_{55}$) DENs before and after UV/O_3 dendrimer removal procedure, Figure S3.
5 CVs of CO electrooxidation on PPF/G6NH$_2$(Au$_{147}$) and PPF/G6OH(Pt$_{55}$) before and after UV/O_3 dendrimer removal procedure, Figure S4.
6 CV overlay of Fc(MeOH)$_2$ redox probe at PPF, PPF/TiO$_{1.9}$ and PPF/TiO$_{2.0}$ working electrodes, Figure S5.
7 CVs of ORR on PPF/TiO$_{1.9}$(2.3nm)/Au$_{147}$ immediately after CO electrooxidation, Figure S6.
8 CVs of CO electrooxidation on PPF/TiO$_x$/Au$_{147}$ electrocatalysts with varying TiO$_x$ thin film thicknesses, Figure S7.
9 CV overlay of Fc(MeOH)$_2$ redox probe at PPF, and PPF/Al$_2$O$_3$ working electrodes, Figure S8.
10 CVs of CO electrooxidation on PPF/Al$_2$O$_3$/Pt$_{55}$ electrocatalysts with different Al$_2$O$_3$ thin film thicknesses, Figure S9.
11 CV overlay of pH 3.1 phosphate buffer at PPF/TiO$_{1,9}$, and PPF/TiO$_{2.0}$ working electrodes, Figure S10.

Deposition of ALD thin films on PPF electrodes. The metal oxide thin films were deposited using a Savannah S100 Cambridge NanoTech ALD system (Ultratech, San Jose, CA). For deposition of the TiO_x thin films, the titanium precursor, TDMAT, was heated to 75 °C, and the oxygen source (H_2O) was maintained at room temperature (22 °C – 25 °C). The TiO_x thin films were deposited using a previously published procedure [10]. Briefly, an ALD cycle was carried as follows: (1) 0.10 s pulse of TDMAT; (2) 20.0 s purge with N_2; (3) 0.015 s pulse of H_2O; and (4) 20.0 s purge with N_2. These four steps were repeated until the desired number of cycles were completed. This procedure results in formation of TiO_x films having a Ti:O ratio of 1.0:1.9 ($TiO_{1.9}$) [10]. To obtain stochiometric $TiO_{2.0}$, the as-deposited films were further oxidized by heating in a tube furnace at 200 °C for 2 h under a constant flow of O_2 (60 sccm).

For deposition of Al_2O_3 thin film, the aluminum precursor, TMA, and the oxygen precursor (O_3) were maintained at room temperature (22 °C – 25 °C). The Al_2O_3 thin films were deposited using a previously published procedure [7]. Briefly, an ALD cycle was carried as follows: (1) 0.015 s pulse of TMA; (2) 20.0 s purge with N_2; (3) 0.015 s pulse of O_3; and (4) 20.0 s purge with N_2. These four steps were repeated until the desired number of cycles were completed.

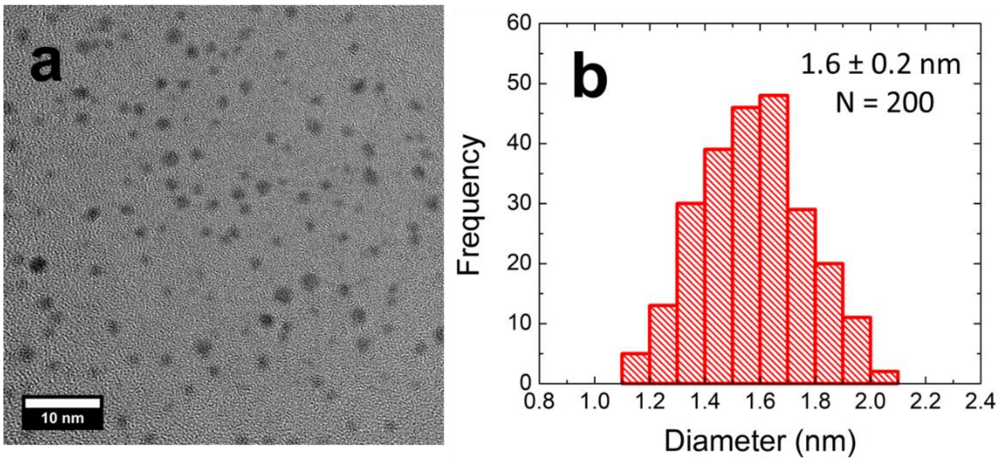

Figure S1. (a) Representative transmission electron micrograph and (b) corresponding particle-size distribution histogram for $G6NH_2(Au_{147})$ DENs.

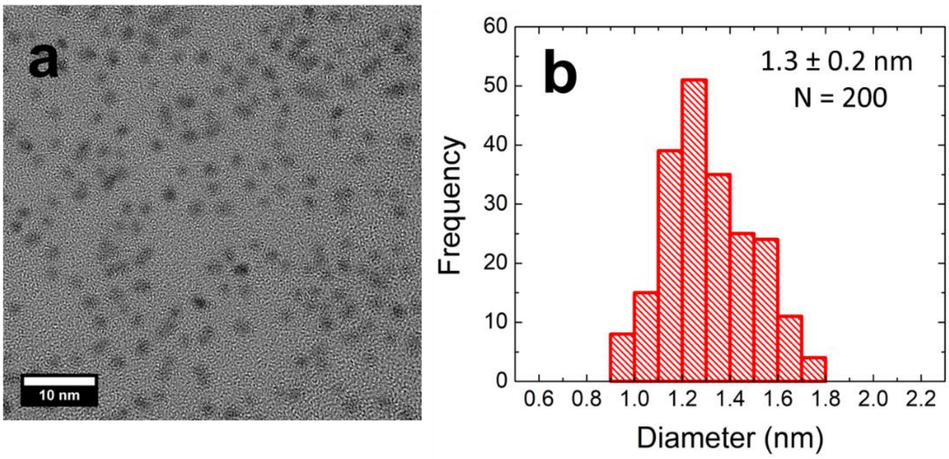

Figure S2. (a) Representative transmission electron micrograph and (b) corresponding particle-size distribution histogram for $G6OH(Pt_{55})$ DENs.

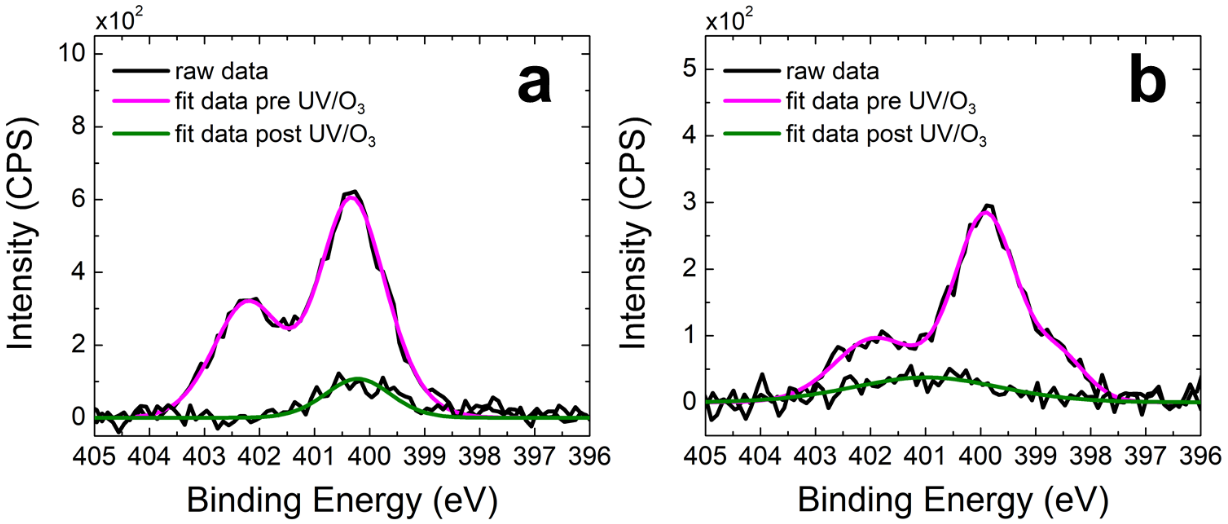

Figure S3. Representative high-resolution XPS spectra in the N 1s regions for (a) PPF/TiO_x(2.3 nm)/$G6NH_2(Au_{147})$ and (b) PPF/TiO_x(2.3 nm)/G6OH(Pt_{55}) before and after the UV/O_3 dendrimer removal procedure. All XPS peaks were referenced to the position of the PPF C 1s peak at 284.50 eV [33]. The XPS measurements were performed within the same area of each support before and after the UV/O_3 dendrimer removal procedure. Following the removal procedure, the dendrimer N 1s signal for $G6NH_2(Au_{147})$ decreases by 89±1% and for G6OH(Pt_{55}) by 80±3%. These values are consistent with our previous reports [7,10]. The XPS measurements were carried out on three independently prepared supports for each modification to ensure reproducibility.

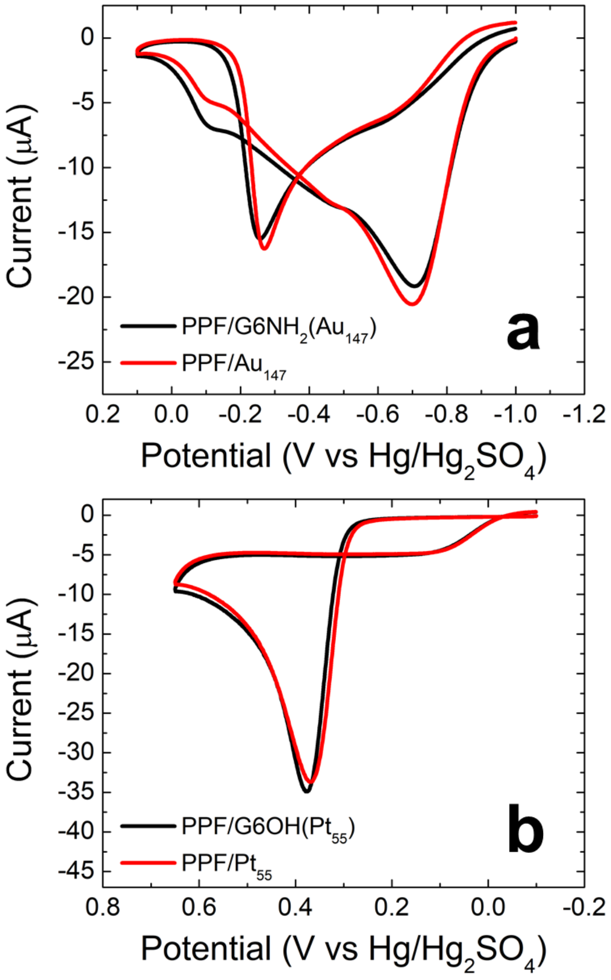

Figure S4. CVs of CO electrooxidation on (a) PPF/$G6NH_2(Au_{147})$ in CO-saturated 0.10 M NaOH and (b) PPF/G6OH(Pt_{55}) in CO-saturated 0.10 M $HClO_4$ before and after the UV/O_3 dendrimer removal procedure. The geometric area of each electrode was 12.4 mm^2. The scan rate was 0.050 V/s. On the

basis of these data, we conclude that there is no significant difference in the CO electrooxidation peak position before and after UV/O_3 dendrimer removal procedure for Au or PtNPs. The experiments were carried out in triplicate on independently prepared electrodes for each surface modification to ensure reproducibility.

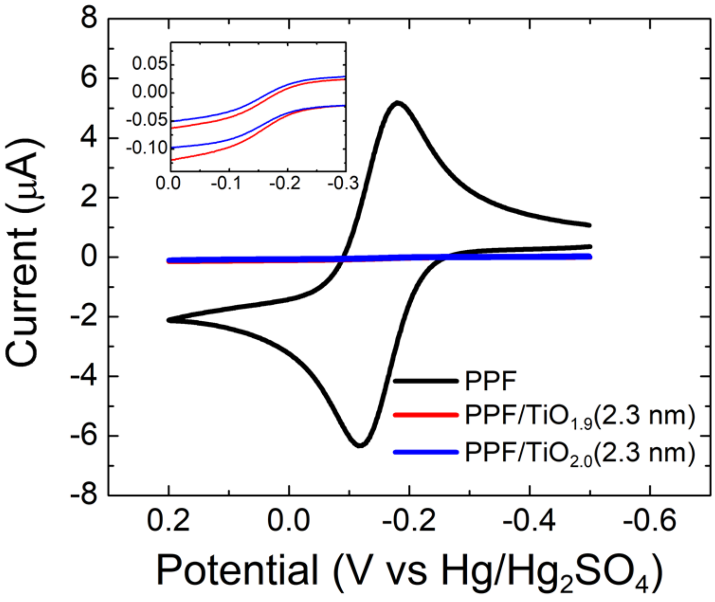

Figure S5. CVs obtained using the electrode configurations shown in the legend. The solutions contained aqueous 1.0 mM $Fc(MeOH)_2$ and 0.10 M KNO_3, the electrode potential was scanned between -0.50 and 0.20 V at 0.010 V/s. The geometric area of each electrode was 12.4 mm^2. The inset shows an expanded view of the potential region where current arising from $Fc(MeOH)_2$ is expected. Electrode passivation is observed for ~2.3 nm-thick TiO_x films atop the PPF electrode regardless of the TiO_x oxidation state. The experiments were carried out in triplicate on independently prepared electrodes for each surface modification to ensure reproducibility.

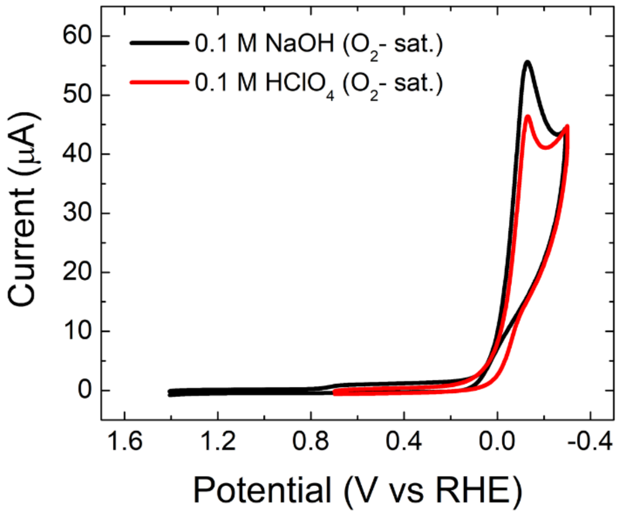

Figure S6. CVs of the ORR on exactly the same electrode, PPF/$TiO_{1.9}$(2.3nm)/Au_{147}, as in **Figure 2a** of the main text. The experiments were carried out immediately after CO electrooxidation measurements described in the *section 3.3* of the main text. The solutions contained O_2-saturated 0.1 M NaOH or 0.1 M $HClO_4$. For the experiment under alkaline conditions, the electrode potential was scanned between 1.4 and -0.2 V vs RHE at 0.050 V/s. For the experiment under acidic conditions, the electrode potential was scanned between 0.7 and -0.2 V vs RHE at 0.050 V/s. The geometric area of each electrode was 12.4 mm^2. The significant ORR activity indicates that the electrocatalyst does not experience electrocatalytic deactivation under CO electrooxidation conditions.

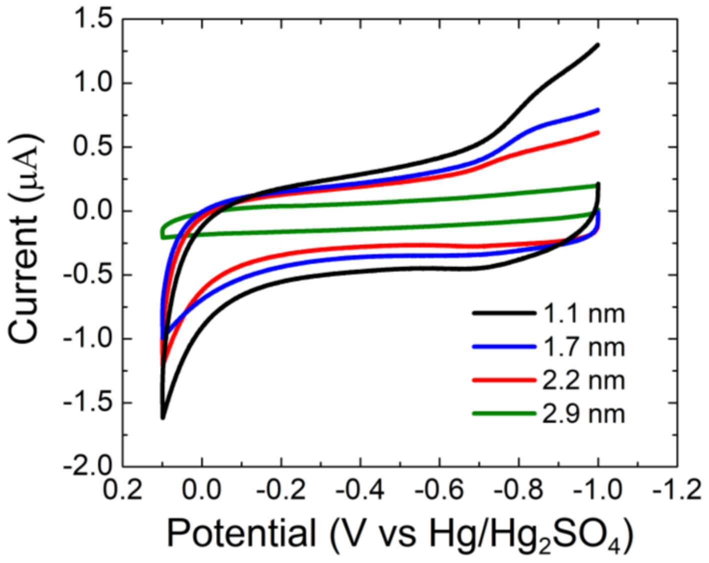

Figure S7. CVs of CO electrooxidation on PPF/TiO_x/Au_{147} electrocatalysts having the TiO_x thicknesses indicated in the legend. The solution contained CO-saturated 0.10 M NaOH. The electrode potential was scanned between -1.0 and 0.10 V at 0.050 V/s. The geometric area of each electrode was 12.4 mm^2. The CV overlay demonstrates the effect of TiO_x thin film thickness on CO electrooxidation activity of the PPF/TiO_x/Au_{147} electrocatalyst. No significant CO electrooxidation activity is observed at any of the TiO_x thicknesses tested. The experiments were carried out in triplicate on independently prepared electrodes for each surface modification to ensure reproducibility.

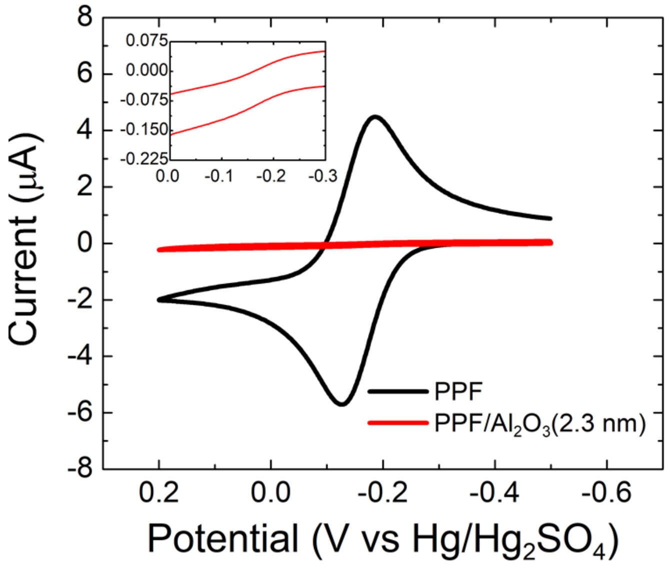

Figure S8. CVs obtained using the electrode configurations shown in the legend. The solutions contained aqueous 1.0 mM $Fc(MeOH)_2$ and 0.10 M KNO_3, the electrode potential was scanned between -0.50 and 0.20 V at 0.010 V/s. The geometric area of each electrode was 12.4 mm^2. The inset shows an expanded view of the potential region where current arising from $Fc(MeOH)_2$ is expected. Electrode passivation is observed for ~2.3 nm-thick Al_2O_3 films atop the PPF. The experiments were carried out in triplicate on independently prepared electrodes for each surface modification to ensure reproducibility.

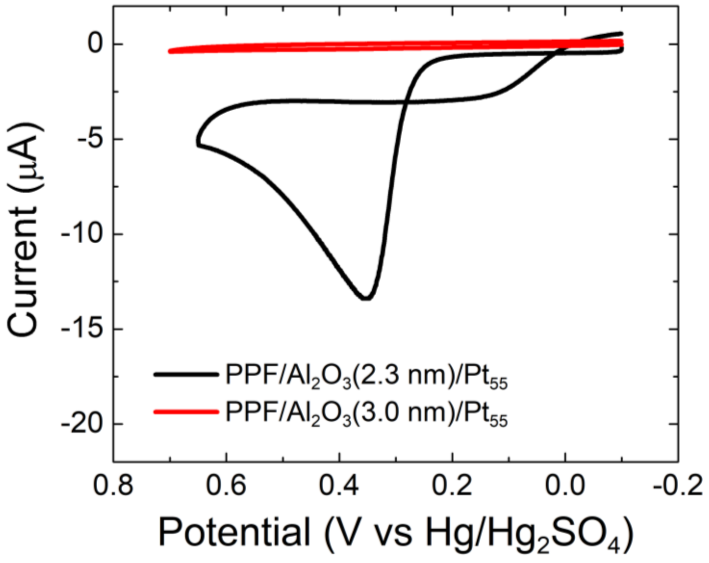

Figure S9. CVs of CO electrooxidation on PPF/Al_2O_3/Pt_{55} electrocatalysts having different Al_2O_3 the thicknesses indicated in the legend. The solution contained CO-saturated 0.10 M $HClO_4$. The electrode potential was scanned between -0.1 and 0.65 V at 0.050 V/s. The geometric area of each electrode was 12.4 mm^2. The CV overlay demonstrates the effect of Al_2O_3 thin film thickness on CO electrooxidation activity of the PPF/Al_2O_3/Pt_{55} electrocatalyst. The experiments were carried out in triplicate on independently prepared electrodes for each surface modification to ensure reproducibility.

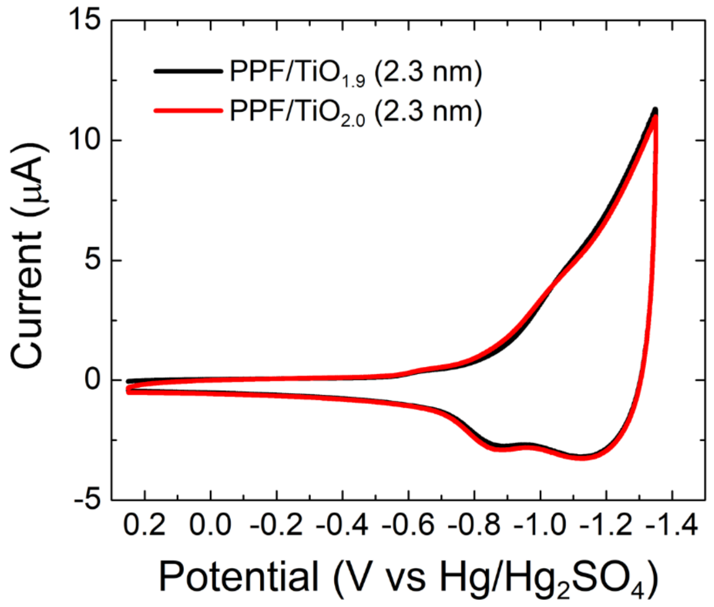

Figure S10. CVs obtained using the electrode configurations shown in the legend. The solutions contained aqueous Ar-saturated 0.10 M phosphate buffer at pH 3.1. The electrode potential was scanned between 0.25 and -1.30 V at 0.050 V/s. The geometric area of each electrode was 12.4 mm^2. The CV overlay demonstrates the background activity for PPF/TiO_x electrodes in the absence of the $Ru(NH_3)_6^{3+}$ redox probe in the buffered solution. The background activity between -0.80 and -1.2 V is attributed to the presence of phosphates, and current increase at -1.3 V arises from the hydrogen evolution reaction [27]. The experiments were carried out in triplicate on independently prepared electrodes for each surface modification to ensure reproducibility.
